# The presence of the reverse distance effect depends on the familiarity of the sequences being processed

**DOI:** 10.1007/s00426-025-02090-8

**Published:** 2025-02-28

**Authors:** Declan Devlin, Korbinian Moeller, Iro Xenidou-Dervou, Bert Reynvoet, Francesco Sella

**Affiliations:** 1 Centre for Mathematical Cognition, School of Science, Loughborough University, Leicestershire, LE11 3TU UK; 2 LEAD Graduate School and Research Network, University of Tübingen, Tübingen, Germany; 3 Brain & Cognition, KU Leuven, Louvain, Belgium; 4 Faculty of Psychology and Educational Sciences, KU Leuven, Kortrijk, Belgium

## Abstract

Number order processing is thought to be characterised by a reverse distance effect whereby consecutive sequences (e.g., 1–2–3) are processed faster than non-consecutive sequences (e.g., 1–3–5). However, there is accumulating evidence that the reverse distance effect is not consistently observed. In this context, the present study investigated whether the presence of the reverse distance effect depends on the familiarity of the sequences being processed. Supporting this proposal, Experiment 1 found that the reverse distance effect was only present when the presented consecutive sequences were considerably more familiar than the presented non-consecutive sequences. Additionally, the sequence 1–2–3 has been suggested to play a pivotal role in the presence of the reverse distance effect due to being both the most familiar and fastest processed sequence. However, it is contested whether 1–2–3 is processed fast because it is familiar or simply because it can typically be verified as ordered from only its first two digits. Supporting the familiarity explanation, Experiments 2 and 3 found that 1–2–3 was processed characteristically fast regardless of whether it could be verified from its first two digits. Taken together, these findings suggest that sequence familiarity plays a critical role in the presence or absence of the reverse distance effect.

**Supplementary Information:**

The online version contains supplementary material available at 10.1007/s00426-025-02090-8.

## Introduction

Order processing is thought to play a prominent role in numerical development. For instance, the faster a person can verify whether or not a number sequence (e.g., 1–2–3) is in order, the better they are likely to perform on an arithmetic task (e.g., Attout et al., 2014; Goffin & Ansari, 2016; Lyons & Beilock, 2011; Lyons et al., 2014; Xu et al., 2023). Moreover, deficits in this order verification capacity are frequently observed in individuals with mathematical learning disabilities (e.g., Decarli et al., 2023; Morsanyi et al., 2018). Accordingly, identifying the strategies and mechanisms underlying order verification performance may be instructive for understanding order processing’s role in numerical development (Devlin et al., 2022).

To investigate these strategies and mechanisms, we can consider how order verification performance varies based on the specific sequences being processed. For instance, ordered sequences (e.g., 1–2–3) are typically processed faster than non-ordered sequences (e.g., 1–3–2) (e.g., Orrantia et al., 2019; Sommerauer et al., 2020), ordered consecutive sequences (e.g., 1–2–3) are typically processed faster than ordered non-consecutive sequences (e.g., 1–3–5) (e.g., Goffin & Ansari, 2016; Lyons & Ansari, 2015; Lyons & Beilock, 2013), ordered ascending sequences (e.g., 1–2–3) are typically processed faster than ordered descending sequences (e.g., 3–2–1) (e.g., Vos et al., 2017; Wong et al., 2022), and ordered regularly spaced sequences (e.g., 2–4–6) are typically processed faster than ordered irregularly spaced sequences (e.g., 2–4–7) (e.g., Vos et al., 2021). It is therefore plausible that such faster-processed sequences are processed using different strategies than such slower-processed sequences (Devlin et al., 2022).

One interpretation of this is that faster-processed sequences are predominantly processed using fast memory-retrieval strategies, whereas slower-processed sequences are predominantly processed using slower alternative strategies, such as sequential comparison (e.g., 5 > 3 and 3 > 1 when processing the triplet 5–3–1) (Devlin et al., 2022; Vos et al., 2021). This is because these fast-processed sequence types are assumed to be more familiar and thus more likely to be retrieved from long-term memory than these slower-processed sequence types. For example, ordered, consecutive, ascending, and regularly spaced sequences are arguably more frequently encountered than non-ordered, non-consecutive, descending, and irregularly spaced sequences. Consistent with this, Dubinkina and colleagues (2021) found that memory-retrieval strategies were self-reported more often when processing ascending or consecutive sequences than when processing descending or non-consecutive sequences. Therefore, it is plausible that faster response times for certain sequence types may be mediated by familiarity and strategy selection.

Although this familiarity perspective may be viewed as an account of order verification performance in general, it is often applied more specifically to explain the reverse distance effect – the finding that consecutive sequences (e.g., 1–2–3) are typically processed faster than non-consecutive sequences (e.g., 1–3–5) (Vos et al., 2017). The reverse distance effect is often viewed as a central characteristic of order verification performance (e.g., Wong et al., 2022). Therefore, explaining its origin may be instructive for identifying the mechanisms underlying order verification itself (Devlin et al., 2022). From the familiarity perspective, the presence of the reverse distance effect may be interpreted simply as a by-product of a more general familiarity effect. That is, consecutive sequences are processed faster because they are more familiar; thus more likely to be processed using memory-retrieval strategies, and more easily retrieved from memory whenever such strategies are applied (Devlin et al., 2024; Vos et al., 2017, 2021).

An alternative explanation, however, is that the reverse distance effect is inherently about consecutiveness. For instance, it has been argued that the reverse distance effect results primarily from the processing of non-consecutive sequences being inhibited, rather than from consecutive sequences being facilitated (e.g., Gattas et al., 2021). This inhibition is thought to result from a conflict with an early-formed intuition that “in-order” refers only to sequences that match the count-list (e.g., 1–2–3, 2–3–4, 3–4–5, and so on). This explanation is plausible since many children appear to possess such an intuition. For example, many children have been found to consistently reject non-consecutive sequences during order verification tasks, suggesting they may not consider them as correctly ordered (Gilmore & Batchelor, 2021; Hutchison et al., 2022; see also Slipenkyj et al., 2024). Moreover, evidence of this count-list intuition has even been observed in an adult population (Gattas et al., 2021; but see Devlin et al., 2023). Therefore, establishing whether the reverse distance effect results from this count-list intuition or from a familiarity effect is critical for identifying the mechanisms underlying order verification performance.

In addition to establishing what drives the presence of the reverse distance effect, it is also necessary to consider what causes absences of this effect. In fact, in recent years, the reverse distance effect has been found to be frequently absent at the individual level. For instance, Sasanguie and Vos (2018) observed no reverse distance effects in both the majority of their first grade participants and approximately half of their second grade participants. Similarly, Vogel and colleagues (2021) observed reverse distance effects in less than half (i.e., 42%) of their adult participants. Moreover, although the reverse distance effect is seemingly more stable at the group level, group-level absences have also been observed (i.e., Brunner et al., 2024; Vos et al., 2021). Accordingly, to provide a coherent account of the mechanisms underlying order verification performance, it is necessary to explain what drives both the presence as well as the absence of the reverse distance effect (Devlin et al., 2022).

Devlin et al. (2024) attempted to explain these absences by reconceptualising the reverse distance effect as a specific instance of a more general familiarity effect. Under this view, although more familiar sequences are expected to be processed faster than less familiar sequences regardless of their distance, consecutive sequences are only expected to be processed faster than non-consecutive sequences when they are also more familiar. Consequently, most people would be expected to produce both a familiarity effect and a reverse distance effect because consecutive sequences are generally more familiar than non-consecutive sequences. However, some individual absences of the reverse distance effect would not be surprising as not all consecutive sequences are necessarily more familiar than all non-consecutive sequences. For example, 2–4–6 and 3–6–9 are arguably highly familiar despite being non-consecutive. Therefore, it is plausible that an individual may not present a reverse distance effect while still exhibiting a familiarity effect.

To test this proposal, Devlin et al. (2024) used a comparative judgement approach to develop a measure of sequence familiarity independent of consecutiveness. This involved participants repeatedly comparing pairs of sequences and choosing the ones they considered to be more familiar from each pair. Notably, although consecutive sequences were generally judged as more familiar than non-consecutive sequences, there were exceptions to this. For example, 1–3–5, 2–4–6, and 3–6–9 were all judged as highly familiar despite being non-consecutive. Moreover, these familiar non-consecutive sequences all elicited correspondingly fast response times. Crucially, although many participants (i.e., 29%) did not display a reverse distance effect, each and every participant appeared to display a familiarity effect (Devlin et al., 2024). In other words, although participants did not always process consecutive sequences faster than non-consecutive sequences, all participants processed familiar sequences faster than unfamiliar sequences. Accordingly, reconceptualising the reverse distance effect as a specific instance of a familiarity effect could plausibly account for individual absences of the reverse distance effect.

Vos and colleagues (2021) proposed a similar familiarity argument for why they did not observe group-level reverse distance effects in their study. In particular, they highlighted how the diverse set of sequences included in their order verification task led to certain highly familiar consecutive sequences (i.e., 1–2–3 and 2–3–4) being left out. As a result, their consecutive sequences were collectively less familiar than in a typical order verification task. Moreover, certain familiar non-consecutive sequences (e.g., 2–4–6) were still included. Arguably, therefore, this resulted in familiarity being relatively balanced across their consecutive and non-consecutive sequences. Accordingly, from a familiarity perspective, the group-level absence of the reverse distance effect in Vos et al. (2021) is consistent with the proposal that one should only expect to observe a reverse distance effect when the included consecutive sequences are collectively more familiar than the included non-consecutive sequences (Devlin et al., 2022; Vos et al., 2021).

The present study aimed to test this hypothesis experimentally. To do this, our first experiment used the familiarity measure developed by Devlin et al. (2024) to create two order verification task conditions: one including the three most familiar consecutive sequences and the three least familiar non-consecutive sequences (i.e., enhanced familiarity condition), and one including the three least familiar consecutive sequences and the three most familiar non-consecutive sequences (i.e., balanced familiarity condition). This resulted in a large difference in familiarity between the consecutive and non-consecutive sequences in the enhanced familiarity condition and only a minimal difference in the balanced familiarity condition. Accordingly, if the reverse distance effect is inherently about consecutiveness, then one would expect consecutive sequences to be processed faster than non-consecutive sequences in both conditions. However, if the presence of the reverse distance effect is dependent on the familiarity of the included sequences, then one would expect to observe a reverse distance effect only in the enhanced familiarity condition, but not in the balanced familiarity condition.

An additional implication of this familiarity perspective is that the presence of the reverse distance effect may be strongly influenced by a few highly familiar consecutive sequences such as 1–2–3. For instance, the omission of just 1–2–3 and 2–3–4 appeared sufficient to eliminate group-level reverse distance effects in the study by Vos et al. (2021). Similarly, Brunner and colleagues (2024) recently replicated such a group-level absence of the reverse distance effect simply by excluding the sequence 1–2–3. Accordingly, highly familiar consecutive sequences such as 1–2–3 appear to play an influential role in the presence and absence of the reverse distance effect.

This seemingly pivotal role of 1–2–3 in the presence of the reverse distance effect is perhaps unsurprising given that 1–2–3 has repeatedly been demonstrated to be the fastest processed sequence (Devlin et al., 2024; Sella et al., 2020). As such, it is inevitable that excluding 1–2–3 from an order verification task would increase overall response times for consecutive sequences and thus reduce the strength of the reverse distance effect. Nonetheless, this influence of 1–2–3 is consistent with the familiarity perspective because, in addition to being the fastest processed sequence, 1–2–3 was also judged by participants as being the most familiar (Devlin et al., 2024). Accordingly, from the familiarity perspective, one would expect the most familiar sequence to also be the fastest processed and thus, resultantly, to play an influential role in the presence and absence of the reverse distance effect.

A key limitation of this argument, however, is that 1–2–3 is not necessarily processed fast *because* it is familiar. Instead, this finding may result from 1–2–3 being able to be determined as ordered after processing only its first two digits. This is because, in typical order verification tasks, no digits smaller than “1” are considered and no digits are ever repeated within a single sequence. Consequently, the third digit of a sequence beginning “1–2” will necessarily be greater than two; therefore, the sequence will necessarily be in order. In fact, this property of 1–2–3 was indicated as the reason for why Brunner and colleagues (2024) excluded 1–2–3 from their order verification task. Crucially, therefore, explaining group-level absences of the reverse distance effect also requires determining why 1–2–3 is processed exceptionally fast. That is, is 1–2–3 processed fast because it is highly familiar, or simply because it can be verified after processing only its first two digits?

Accordingly, the second and third experiments of this study aimed to differentiate between these two explanations. In Experiment 2, participants completed two order verification task conditions: one including sequences made from the digits 1–9 and one including sequences made from the digits 0–8. In the 1–9 condition, 1–2–3 could still be verified as ordered after processing its first two digits. However, in the 0–8 condition, all three digits necessarily needed to be processed for 1–2–3 to be verified as ordered; this is because 1–2–0 was a possible sequence in this condition. Accordingly, if 1–2–3 is processed fast because it is familiar, it would be expected to be processed comparably fast in the 1–9 and 0–8 conditions. Alternatively, if 1–2–3 is only processed fast because it can be verified after its first two digits, it would be expected to be processed faster in the 1–9 condition than in the 0–8 condition.

Furthermore, in the 0–8 condition, the sequence 0–1–2 was able to be verified as ordered from its first two digits while 1–2–3 was not. This is because there were no digits smaller than “0” and no digits were repeated within a single sequence; therefore, any sequence beginning with “0–1” would necessarily be in order. Nonetheless, 0–1–2 is arguably a relatively unfamiliar sequence – at least compared to 1–2–3. Accordingly, if fast response times reflect high familiarity, then 1–2–3 would be expected to be processed faster than 0–1–2. In contrast, if fast response times result from being able to verify a sequence after its first two digits, then 0–1–2 would be expected to be processed faster than 1–2–3.

Finally, Experiment 3 involved another two order verification task conditions: one in which no digits were repeated within a sequence, and another in which digits were sometimes repeated (e.g., 3–4–3). In the standard condition, therefore, participants could still verify 1–2–3 as ordered after its first two digits. However, in the repeated digits condition, all three digits necessarily needed to be processed before 1–2–3 could be verified as ordered, since 1–2-1 was a possible sequence in this condition. Accordingly, if 1–2–3 is processed fast because of its high familiarity, it should be processed comparably fast in the standard and repeated digits conditions. Alternatively, if 1–2–3 is only processed fast because it can be verified after its first two digits, then it would be expected to be processed faster in the standard condition than in the repeated digits condition.

## Experiment 1: Method

Experiment 1 was pre-registered using AsPredicted (aspredicted.org/Z95_XSY). All three experiments in this study received approval from Loughborough University’s ethics committee. Data, materials, and analysis scripts for all three experiments are accessible via the Open Science Framework: osf.io/djsxf.

### Participants

One hundred UK-based participants (mean age = 40.57 years, *SD* = 12.79) were recruited online via Prolific.com. Each participant received £2.00 for participating. This number of participants was pre-registered based on an a priori estimation of required sample size which indicated that with an alpha of 0.05 and power set to 0.95, a minimum of 59 participants would be needed to detect a moderate effect size (f^2^ = 0.15) in a 2 × 2 repeated measures ANOVA.

### Tasks

Participants completed two order verification task conditions (enhanced familiarity condition, balanced familiarity condition) and an arithmetic verification task. The arithmetic task was administered only for exploratory purposes; all details of this task and the respective analyses are provided in the supplementary materials. The two order verification task conditions were always completed first, in a counterbalanced order, followed by the arithmetic verification task. Tasks for all three experiments were programmed using PsychoPy (Peirce et al., 2019) and presented to participants via Pavlovia.org.

#### Order verification task

**Fig. 1:**
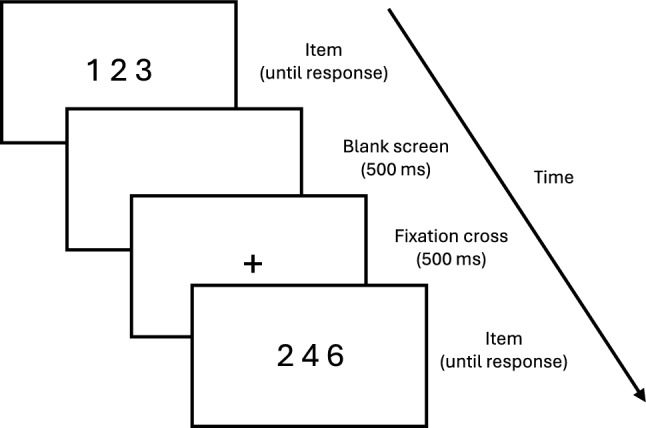
Example and timings of the order verification task

The design of the order verification task was based on tasks used in previous studies (e.g., Bourassa, 2014; Dubinkina et al., 2023; Morsanyi et al., 2024). Three-digit sequences (e.g., 1–2–3) were presented in the centre of the screen and participants had to indicate whether they were in order or not using the “P” and “Q” keys on a standard QWERTY keyboard. Here, “P” always indicated “in order”, and “Q” always indicated “not in order”. Sequences remained on screen until a key press was registered. This was followed by a blank screen, a fixation cross, and then the next sequence (see Fig. 1 for example and timings).

Sequences for both conditions were selected using the familiarity scores generated by Devlin et al. (2024). For simplicity, only ascending sequences were considered. Additionally, all ordered sequences were regularly spaced and composed of three single digits. The enhanced familiarity condition included the three most familiar consecutive sequences (mean familiarity score = 81.02) and the three least familiar non-consecutive sequences (mean familiarity score = 17.47) (see Table 1). The balanced familiarity condition included the three least familiar consecutive sequences (mean familiarity score = 48.58) and the three most familiar non-consecutive sequences (mean familiarity score = 46.15) (see Table 2). In both conditions, each ordered sequence also had a corresponding non-ordered sequence (e.g., 1–2–3 and 1–3–2).

**Table 1:** All unique sequences in the enhanced familiarity condition

Left	Centre	Right	Type	Familiarity score
1	2	3	Consecutive	100
2	3	4	Consecutive	78
3	4	5	Consecutive	65.06
1	3	2	Non-ordered	–
2	4	3	Non-ordered	–
3	5	4	Non-ordered	–
1	4	7	Non-consecutive	21.56
2	5	8	Non-consecutive	17.22
5	7	9	Non-consecutive	13.64
1	7	4	Non-ordered	–
2	8	5	Non-ordered	–
5	9	7	Non-ordered	–

**Table 2:** All unique sequences in the balanced familiarity condition

Left	Centre	Right	Type	Familiarity score
5	6	7	Consecutive	49.67
7	8	9	Consecutive	49.60
6	7	8	Consecutive	46.46
5	7	6	Non-ordered	–
7	9	8	Non-ordered	–
6	8	7	Non-ordered	–
2	4	6	Non-consecutive	51.44
1	3	5	Non-consecutive	43.75
3	6	9	Non-consecutive	43.25
2	6	4	Non-ordered	–
1	5	3	Non-ordered	–
3	9	6	Non-ordered	–

In each condition, each unique sequence was presented five times, resulting in 60 trials per condition and 120 trials in the full task. Additionally, before starting the task, participants completed ten randomly selected practice trials with on-screen feedback provided. No feedback was given during critical trials. Overall, the order verification task, including both conditions, lasted approximately six minutes.

## Experiment 1: Results

Analyses for all three experiments were conducted using the statistical programming language R within RStudio (R Core Team, 2020; RStudio Team, 2022).

### Order verification performance

We first checked participants’ accuracy on the order verification task. In line with our pre-registered exclusion criteria, seven participants were excluded for either (i) overall accuracy three standard deviations below the mean or (ii) below 70% accuracy on either consecutive or non-consecutive sequences. Additionally, one other participant was excluded for not completing the full task. Consequently, responses from 92 participants were included in the final analysis.

Each participant completed 60 order verification trials per condition, resulting in 11,040 responses collected across 92 participants. Mean accuracy across both conditions was 96.64%. Because we were only interested in correct responses for ordered sequences, we first removed all non-ordered trials (n = 5520; 50%) and all incorrect responses (n = 185; 3.35%). Then, as pre-registered, we removed responses longer than the mean plus three standard deviations (mean = 1239 ms, *SD* = 4473 ms) (n = 2; 0.04%). We note that there were no responses shorter than 200 ms.

Following this trimming procedure, the distribution was still positively skewed (skewness = 4.27) and highly leptokurtic (kurtosis = 46). Therefore, to account for this, we calculated median response times for each participant for each unique sequence. After this, we calculated average median response times and standard deviations for all possible condition (enhanced/balanced) and distance (consecutive/non-consecutive) combinations (see Table 3). This distribution remained non-normally distributed, as indicated by a Shapiro–Wilk test (W = 0.82, *p* < 0.001); therefore, we also report non-parametric results for the main analyses below.

**Table 3:** Means and standard deviations for all condition/distance combinations

Condition	Distance	Average median RT (ms)	*SD*
Enhanced	Consecutive	939	283
	Non-consecutive	1353	588
Balanced	Consecutive	1097	310
	Non-consecutive	1068	361

Next, we evaluated the influences of distance and familiarity on order verification speed. As pre-registered, we conducted a 2 × 2 repeated measures ANOVA with one factor of *distance* (consecutive/non-consecutive) and one factor of *familiarity* condition (enhanced familiarity/balanced familiarity). The ANOVA indicated a significant main effect of distance, with consecutive sequences being processed faster than non-consecutive sequences, *F*(1, 91) = 56.75, *p* < 0.001, $\eta _{p}^{2}$ = 0.38. As well as a significant main effect of condition, with responses being faster in the balanced familiarity condition (mean = 1083 ms, *SD* = 337 ms) than in the enhanced familiarity condition (mean = 1146 ms, *SD* = 506 ms), *F*(1, 91) = 8.29, *p* = 0.005, $\eta _{p}^{2}$ = 0.08.

**Fig. 2:**
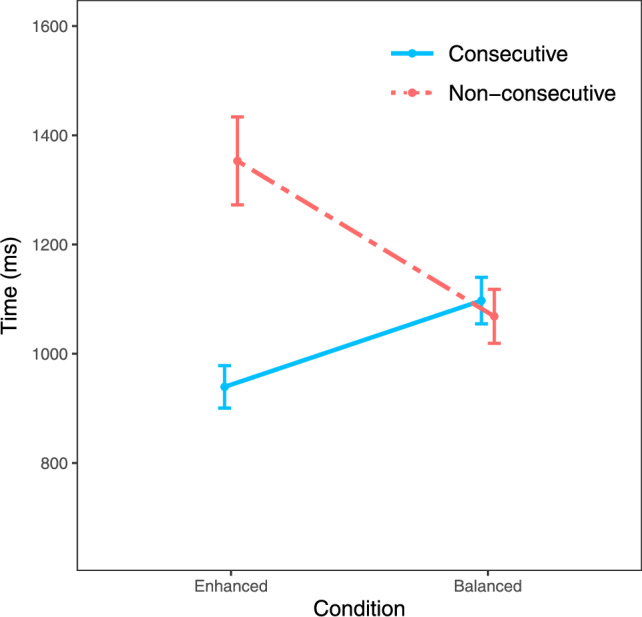
Response times by familiarity condition. Error bars denote 95% confidence intervals

Crucially, as expected, there was a significant interaction between distance and condition, indicating that the reverse distance effect was significantly larger in the enhanced familiarity condition than in the balanced familiarity condition, *F*(1, 91) = 107.12, *p* < 0.001, $\eta _{p}^{2}$ = 0.54. Closer inspection via post-hoc paired-samples *t* tests revealed that although there was a significant reverse distance effect in the enhanced familiarity condition [*t*(91) = 9.77, *p* < 0.001], there was no significant difference in response times between consecutive and non-consecutive sequences in the balanced familiarity condition [*t*(91) = 1.69, *p* = 0.095] (see Fig. 2). Wilcoxon signed-rank tests produced the same pattern of results, indicating a strong reverse distance effect in the enhanced familiarity condition (V = 1, *p*_adj_ < 0.001) and no distance effect in the balanced familiarity condition (V = 2742, *p*_adj_ = 0.133).

**Fig. 3:**
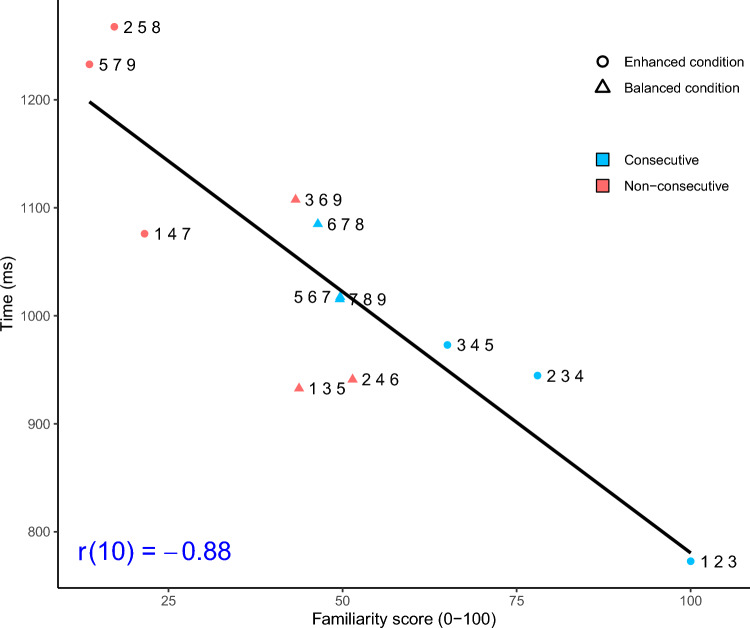
Median response times by familiarity score across both conditions

Notably, familiarity scores for consecutive and non-consecutive sequences were very similar in the balanced familiarity condition (48.48 vs. 46.15). Therefore, observing no distance effect in this condition is consistent with the familiarity perspective. In this context, we also considered the overall association between familiarity scores and median response times across both conditions. When considering both conditions, we observed a very strong negative correlation whereby more familiar sequences were processed faster than less familiar sequences, *r*(10) = –0.88, *p* < 0.001 (see Fig. 3).

## Experiment 2: Method

Experiments 2 and 3 were also pre-registered via AsPredicted (aspredicted.org/V8R_QP5).

### Participants

A separate group of 101 participants (mean age = 40.48 years, *SD* = 14.05) was recruited using Prolific.com. Each participant received £1.20 for participating. A target of 100 participants was pre-registered based on an a priori estimation of required sample size, which indicated that with an alpha of 0.05 and power set to 0.80, a minimum of 90 participants would be needed to detect a small to moderate effect size (Cohen’s *d* = 0.30) using a two-tailed paired samples *t* test. An additional participant was tested due to an error during data collection.

### Tasks

Each participant completed two order verification task conditions (1–9 condition, 0–8 condition) in a counterbalanced order.

#### Order verification task

This order verification task followed the same general design as the task used in Experiment 1 (see Fig. 1 for example). The only notable difference concerns the sequences presented to participants. For simplicity, only ascending consecutive (e.g., 1–2–3) and non-ordered sequences (e.g., 1–3–2) were included in both conditions. In the 1–9 condition, sequences were composed of the digits 1–9. In the 0–8 condition, sequences were composed of the digits 0–8. The range 0–8 was chosen over 0–9 simply to ensure the two conditions were of equal length. The full list of included sequences is provided in Tables S2 and S3 in the supplementary materials.

In both conditions, each unique sequence was presented six times, resulting in 84 trials per condition and 168 trials in total. As a result, the experiment lasted approximately seven minutes.

## Experiment 2: Results

### Order verification performance

We first checked participants’ accuracy across both conditions. In line with our pre-registered criteria, we excluded two participants who displayed overall accuracy more than three standard deviations below the mean. Consequently, responses from only 99 participants were included in the subsequent analysis.

Each participant completed 84 order verification trials per condition, resulting in a total of 16,632 responses recorded across 99 participants. Mean accuracy across both conditions was 97.10%. Because we were only interested in correct responses for ordered sequences, we removed all non-ordered trials (n = 8316; 50%) and all incorrect responses (n = 252; 3.03%). Additionally, as pre-registered, we removed responses that were shorter than 200 ms (n = 2; 0.02%) or longer than the overall mean plus three standard deviations (mean = 943 ms, *SD* = 1,426 ms) (n = 6; 0.07%).

**Fig. 4:**
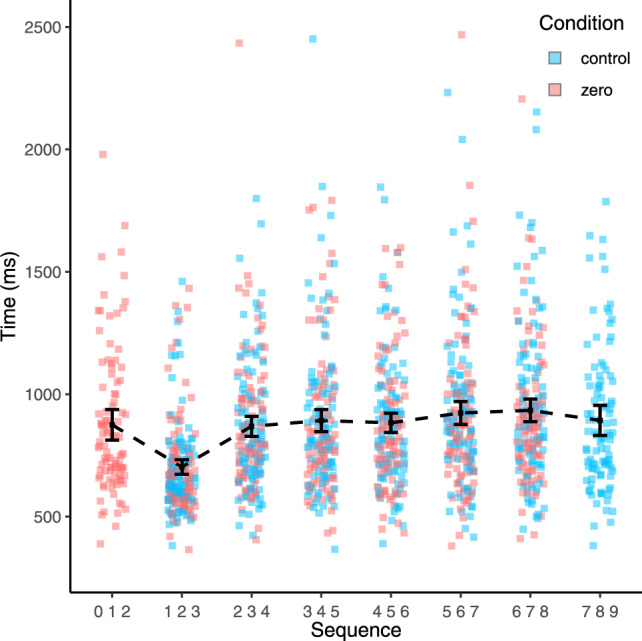
Response times for each sequence across both conditions. Error bars denote 95% confidence intervals

Following this trimming procedure, the distribution still appeared positively skewed (skewness = 3.04) and leptokurtic (kurtosis = 21.93). Therefore, to account for this, we calculated median response times for each participant for each condition. Using these medians, we then calculated the average median response time across all sequences for each condition which was 859 ms (*SD* = 242 ms) for the 1–9 condition and 839 ms (*SD* = 232 ms for the 0–8 condition. Median response times for each sequence across both conditions are displayed in Fig. 4. The median response times remained non-normally distributed as indicated by a Shapiro–Wilk test (W = 0.90, *p* < 0.001); therefore, we also report non-parametric results for the main analyses below.

#### 1–2–3 (1–9) vs. 1–2–3 (0–8)

**Fig. 5:**
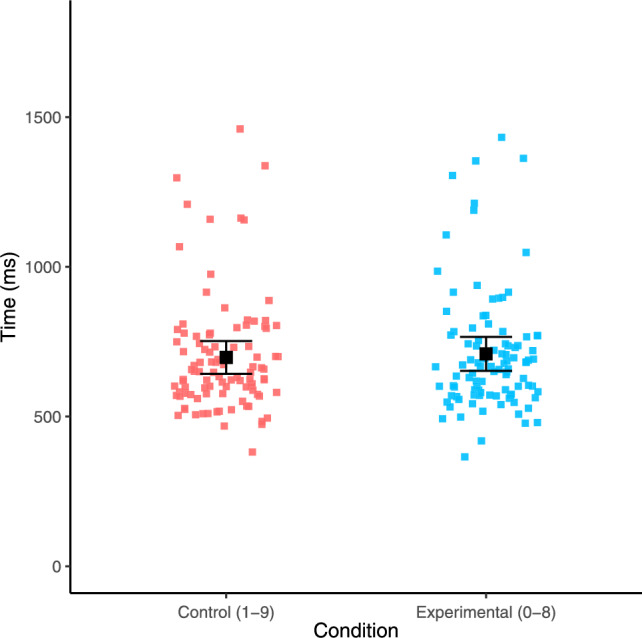
Response times for 1–2–3 in each condition. Error bars denote 95% confidence intervals

We then considered whether response times for the sequence 1–2–3 differed between the 1–9 and 0–8 conditions. To do this, we first calculated median response times for 1–2–3 for each participant for each of the two conditions. Then, as pre-registered, we conducted a paired-samples *t* test that revealed there was no significant difference in response times for 1–2–3 between the 1–9 (mean = 697 ms; *SD* = 195 ms) and 0–8 (mean = 709 ms; *SD* = 202 ms) conditions, *t*(98) = 1.31, *p* = 0.193. This same pattern of results was indicated by a Wilcoxon signed-rank test, V = 2174, *p* = 0.294. These findings are visualised in Fig. 5.

As an additional analysis, we conducted a Bayesian paired-samples *t* test using the BayesFactor (Morey et al., 2022) package in R. This returned a Bayes Factor of 0.25, providing moderate support for the null hypothesis (i.e., no difference in response times between the two conditions) according to the classification by Jeffreys (1961).

#### 0–1–2 vs. 1–2–3

**Fig. 6:**
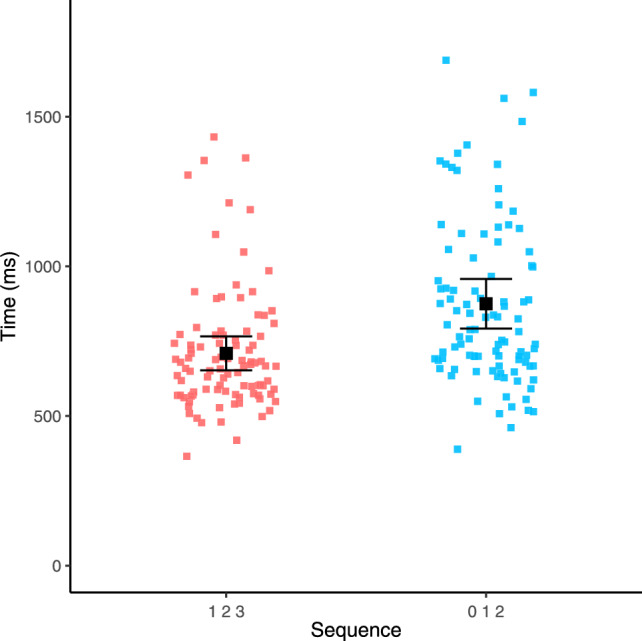
Response times for 1–2–3 and 0–1–2 in the 0–8 condition only. Error bars denote 95% confidence intervals

We next considered whether response times differed between 0–1–2 and 1–2–3 in the 0–8 condition. This was because, in this condition, 0–1–2 could be verified as ordered after only its first two digits whereas 1–2–3 could not. Therefore, we evaluated whether 1–2–3 was processed faster or slower than 0–1–2 in this condition. To do this, we first calculated each participant’s median response times for both 1–2–3 and 0–1–2 in the 0–8 condition only. We then conducted a paired-samples *t* test that indicated that participants were significantly faster at processing 1–2–3 (mean = 709 ms, *SD* = 202 ms) than they were at processing 0–1–2 (mean = 875 ms, *SD* = 294 ms), *t*(98) = 9.13, *p* < 0.001, Cohen’s *d* = 0.59. This same pattern of results was indicated by a Wilcoxon Signed-Rank test, V = 234, *p* < 0.001. Accordingly, this supports the hypothesis that 1–2–3 is processed fast on account of its high familiarity rather than due to its ability to be verified from its first two digits. These results are visualised in Fig. 6.

One possible explanation for this finding is that, because conditions were presented in a counterbalanced order, approximately half of the participants completed the control (1–9) condition before the experimental (0–8) condition. Therefore, these participants would have practiced processing 1–2–3 multiple times before encountering the sequence 0–1–2. Consequently, this practice may have improved performance on 1–2–3 in the 0–8 condition, thus producing the observed pattern of response times.

As an exploratory analysis, we tested this explanation by conducting a mixed factorial ANOVA with a between-participants factor of presentation order (control-then-experimental, experimental-then-control) and a within-participants factor of sequence (0–1–2, 1–2–3). This ANOVA again indicated a main effect of sequence whereby 1–2–3 was processed faster than 0–1–2, *F*(1, 97) = 89.45, *p* < 0.001, $\eta _{p}^{2}$ = 0.480. Furthermore, although there was no significant main effect of presentation order, *F*(1, 97) = 1.23, *p* = 0.270, there was a significant interaction between presentation order and sequence, *F*(1, 97) = 6.53, *p* = 0.012, $\eta _{p}^{2}$ = 0.063.

To better understand this interaction, we conducted post-hoc paired-samples *t* tests separately for both presentation orders. In the control-then-experimental order, response times were still significantly faster for 1–2–3 (mean = 713 ms, *SD* = 203 ms) compared to 0–1–2 (mean = 925 ms, *SD* = 301 ms), *t*(47) = 7.21, *p* < 0.001. Likewise, in the experimental-then-control order, response times were also significantly faster for 1–2–3 (mean = 706 ms, *SD* = 203 ms) compared to 0–1–2 (mean = 828 ms, *SD* = 281 ms), *t*(50) = 6.05, *p* < 0.001. Notably, this difference was larger for the control-then-experimental order (difference = 213 ms) than for the experimental-then-control order (difference = 122 ms).

Interestingly, this interaction appeared to result not from 1–2–3 being processed faster in the second half of the task, but rather from 0–1–2 being processed slower in the second half of the task. This is perhaps unexpected because one may have expected practice effects to result in faster response times for all sequences, including 0–1–2, in the second half of the task. One possible explanation for this finding may be that it was the surprising introduction of the digit “0” only in the second half of the task that resulted in slower performance on 0–1–2 compared to when “0” was simply included at the start of the experiment.

## Experiment 3: Method

Another 100 UK-based participants (mean age = 39.55 years, *SD* = 12.78) were recruited via Prolific.com. Each participant received £1.20 for participating. This number of participants was pre-registered based on the a priori estimation of required sample size conducted for Experiment 2, which indicated that a minimum of 90 participants would be needed to detect a small to moderate effect size (Cohen’s *d* = 0.30) using a two-tailed paired samples *t* test.

### Tasks

Each participant completed two order verification task conditions (control condition, repeated digits condition) in a counterbalanced order.

#### Order verification task

The general format of this task was identical to the order verification task used in Experiment 2. The only changes were to the sequences included in the two conditions. For simplicity, both conditions included only ascending consecutive and non-ordered sequences. In the control condition, sequences were composed of the digits 1–9, with no digits repeated within a single sequence (for full list of sequences, see Table S2 in the supplementary materials). In the repeated digits condition, sequences were again composed of the digits 1–9; however, half of the non-ordered sequences contained repeated digits (e.g., 1–2–1; 2–1–2) (for full list of sequences, see Table S4 in the supplementary materials).

In the control condition, each unique sequence was presented six times. In the repeated digits condition, each unique ordered sequence was also presented six times. However, because all sequences with repeated digits were non-ordered, there were now twice as many unique non-ordered sequences. Therefore, each unique non-ordered sequence was presented only three times in the repeated digits condition to ensure that participants were exposed to 50% ordered and 50% non-ordered sequences in both conditions. Accordingly, there were 84 trials in each condition, and 168 trials in total. Overall, the full task lasted approximately seven minutes.

## Experiment 3: Results

### Order verification performance

We first checked participants’ accuracy across both conditions. In line with our pre-registered criteria, two participants were removed due to displaying overall accuracy more than three standard deviations below the mean. Consequently, responses from only 98 participants were included in the final analysis.

Each participant completed 84 order verification trials per condition, resulting in 16,464 responses recorded across 98 participants. Mean accuracy across both conditions was 96.85%. Because we were only interested in correct responses for ordered sequences, we again removed all non-ordered trials (n = 8232; 50%) and all incorrect responses (n = 286; 3.47%). Additionally, as pre-registered, we removed responses that were shorter than 200 ms (n = 1; 0.01%) or longer than the overall mean plus three standard deviations (mean = 922 ms; *SD* = 504 ms) (n = 103; 1.30%).

**Fig. 7:**
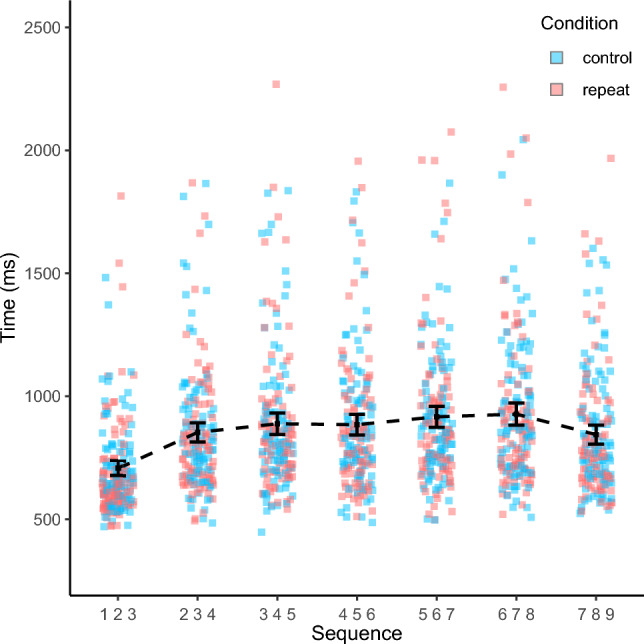
Response times for each sequence across both conditions. Error bars denote 95% confidence intervals

Following this trimming procedure, the distribution was now only slightly positively skewed (skewness = 1.69) but remained leptokurtic (kurtosis = 9.3). Therefore, to account for this, we again calculated median response times for each participant for each condition. Using these medians, we calculated average median response times for each condition: control condition mean = 857 ms (*SD* = 238 ms); repeated digits condition mean = 832 ms (*SD* = 242 ms). Median response times for each sequence across both conditions are displayed in Fig. 7. The distribution of median response times remained non-normally distributed, as indicated by a Shapiro–Wilk test (W = 0.86, *p* < 0.001); therefore, we also report non-parametric results for the analyses below.

#### 1–2–3 (control) vs. 1–2–3 (repeated digits)

We next considered whether response times for the sequence 1–2–3 differed between the control and repeated digits conditions. This was because 1–2–3 could be verified as ordered from its first two digits in the control condition, but not in the repeated digits condition. We first calculated median response times for 1–2–3 for each participant for each of the two conditions. Then, as pre-registered, we conducted a paired-samples *t* test that revealed there was no significant difference in response times for 1–2–3 between the control (mean = 711 ms, *SD* = 179 ms) and repeated digits conditions (mean = 702 ms, *SD* = 214 ms), *t*(97) = 0.76, *p* = 0.447. The same pattern of results was indicated by a Wilcoxon signed-rank test, V = 2774, *p* = 0.218.

**Fig. 8:**
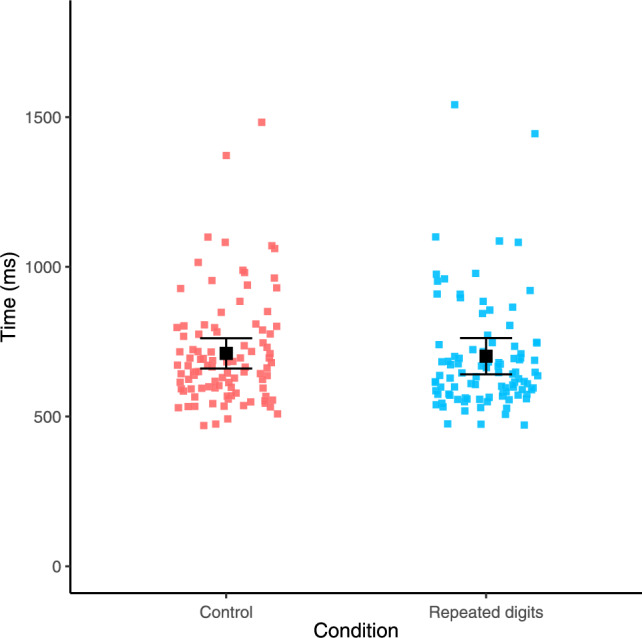
Response times for 1–2–3 in each condition. Error bars denote 95% confidence intervals

Additionally, we again reran this analysis using a Bayesian paired-samples *t* test. This returned a Bayes Factor of 0.15, providing moderate support for the null hypothesis (i.e., no difference in response times between the two conditions), according to the classification by Jeffreys (1961). These findings are visualised in Fig. 8.

## Discussion

This study investigated the role of sequence familiarity in the presence and absence of the reverse distance effect across three experiments. The first experiment tested the proposal that group-level reverse distance effects are only expected when the included consecutive sequences are considerably more familiar than the included non-consecutive sequences. To do this, we compared performance on an order verification task including the most familiar consecutive sequences and the least familiar non-consecutive sequences, to one including the least familiar consecutive sequences and the most familiar non-consecutive sequences. As predicted, we observed a reverse distance effect only in the condition in which the included consecutive sequences were considerably more familiar than the included non-consecutive sequences. Additionally, across both conditions, we observed a strong familiarity effect whereby more familiar sequences were processed faster than less familiar sequences. Accordingly, these findings suggest that sequence familiarity plays an influential role in both the presence and absence of the reverse distance effect.

Importantly, these findings also help differentiate between the familiarity and count-list interpretations of the reverse distance effect. From the familiarity perspective, the reverse distance effect is independent of consecutiveness because not every consecutive sequence is necessarily more familiar than every non-consecutive sequence (Devlin et al., 2024); therefore, the reverse distance effect is expected to vary based on the familiarity of the sequences being processed (Devlin et al., 2022; Vos et al., 2021). Conversely, from the count-list perspective, the reverse distance effect is inherently about consecutiveness because non-consecutive sequences are thought to be processed slower due to conflicting with an intuition that only count-list sequences are correctly ordered (Gattas et al., 2021; Hutchison et al., 2022); therefore, the reverse distance effect should always be expected because non-consecutive sequences necessarily never match the count-list. Accordingly, because we found that consecutive sequences were only processed faster when they were also more familiar, our findings substantiate the view that the reverse distance effect is better characterised as a familiarity effect than as a count-list effect.

Another implication of these findings is that certain highly familiar sequences –such as 1–2–3–may have an outsized impact on the presence or absence of the reverse distance effect. This aligns with previous work suggesting that excluding 1–2–3 from order verification tasks may eliminate group-level reverse distance effects (e.g., Brunner et al., 2024; Vos et al., 2021). It is contested, however, whether 1–2–3 is processed fast because it is familiar, or simply because it can typically be verified as ordered from its first two digits. To address this, we compared performance on a standard order verification task to two alternative versions in which 1–2–3 could only be verified after processing all three digits: one including the digit “0” (Experiment 2) and one including repeated digits (Experiment 3). Notably, in both cases, 1–2–3 was processed comparably fast regardless of whether it could be determined as ordered from its first two digits. Accordingly, these findings suggest that 1–2–3 is processed fast due to its high familiarity, rather than because of any such incidental property of the task.

One could argue, however, that adding the digit “0” to the order verification task was a very subtle manipulation that may not have been especially noticeable to participants. For instance, in the “0” digit condition, only two of the fourteen sequences (i.e., 14.29%) contained a zero (i.e., 1–2–0 and 0–1–2). Consequently, some participants may not have noticed when “0” was included and thus still applied the heuristic that any sequence beginning with “1–2” must necessarily be ordered, even when this was not the case. Notably, however, 1–2–3 was also processed characteristically fast in the repeated digits condition in which the manipulation was much more apparent. For instance, in that condition, 25% of the presented sequences contained repeated digits. Therefore, it is unlikely the present findings can be explained by participants simply failing to notice when zeros or repeated digits were included in the task.

Another key finding from the “0” digit condition was that participants processed 1–2–3 faster than 0–1–2. This is notable because, although 1–2–3 could no longer be verified from its first two digits, 0–1–2 could now be verified from *its* first two digits. Accordingly, if order verification performance was strongly influenced by this incidental property of the task, 0–1–2 would have been expected to be processed faster than 1–2–3 in this condition. Instead, because 0–1–2 is arguably relatively unfamiliar compared to 1–2–3, this finding further strengthens the view that order verification performance is driven primarily by the familiarity of the sequences being processed.

It should be noted, however, that although the present findings suggest the fast processing of 1–2–3 likely does not result from its ability to be verified from its first two digits, this does not necessarily rule out other alternative explanations. For instance, one possibility is that 1–2–3 is processed fast due to a semantic end effect whereby processing is enhanced when one of the involved stimuli is the smallest value in the set (Pinhas & Tzelgov, 2012; Pinhas et al., 2015). For example, magnitude is compared faster for pairs such as 1 and 3 than 3 and 5 (e.g., Banks, 1977; Leth-Steensen & Marley, 2000). In the context of order, “1” is arguably the smallest value as “0” has little ordinal meaning in everyday life. Therefore, the faster processing of 1–2–3 than 0–1–2 in Experiment 2 does not rule out 1–2–3 being processed faster due to a semantic end effect. However, although an end effect could plausibly explain why 1–2–3 is processed exceptionally fast, it cannot explain more general familiarity effects, such as 2–4–6 being processed faster than 3–4–5 (e.g., Devlin et al., 2024). Accordingly, more research is needed to differentiate between the familiarity and end effect accounts of why 1–2–3 is processed uniquely fast.

Additionally, although the inclusion of 1–2–1 in Experiment 3 meant that the third digit of a sequence beginning with “1–2” could have been either 3 or 1, this may not have necessarily prevented semi-automatic validation of 1–2–3. For instance, 1–2–1 could have been rejected based on the early recognition of identical symbols at both ends of the sequence. Consequently, in the repeated digits condition, 1–2–3 may have been quickly verified based on (i) its first two digits being “1–2” and (ii) the absence of identical symbols. Therefore, comparable response times for 1–2–3 in the control and repeated digits conditions do not conclusively rule out the possibility that 1–2–3’s fast processing results from this property of the task. To address this, future research could compare performance on a standard order verification task to one including less familiar sequences such as 1–2–4 and 1–2–5. If 1–2–3 is processed faster due to its high familiarity, it should remain the fastest-processed sequence. However, if its fast processing results from its ability to be verified from its first two digits, 1–2–4 and 1–2–5 should elicit comparably fast response times.

The implications of the present findings are particularly relevant given that ascending single-digit triplets are one of the most commonly studied sequence types in the order processing literature. However, future studies may also benefit from considering a broader range of sequence types. For instance, only considering ascending and single-digit sequences arguably limited our ability to differentiate the effects of familiarity from the effects of numerical size. This is because, in the present study, the more familiar sequences tended to have a smaller numerical size. For example, 1–2–3 and 1–3–5 were classified as familiar and involved smaller numbers, whereas 5–6–7 and 5–7–9 were classified as unfamiliar and involved larger numbers. Consequently, it is possible that numerical size contributed to the absence of the reverse distance effect observed in Experiment 1.

It is unlikely, however, that numerical size alone produced these findings. For instance, previous work using a linear mixed-effects modelling approach found that adding familiarity scores to a model including consecutiveness, direction, and numerical size improved model fit (Devlin et al., 2024). Moreover, although a model incorporating all of these predictors explained more variance, a familiarity-only model provided the most parsimonious explanation of order verification performance, as indicated by it having the lowest Bayesian Information Criterion value. Consistent with this, the present study found that 1–2–3 was processed faster than 0–1–2 – the only included sequence with a smaller numerical size than 1–2–3 – suggesting that familiarity likely has a stronger influence on order verification performance than numerical size. Nonetheless, future research may better differentiate between the influences of familiarity and numerical size by considering sequences which are both highly familiar and involve large numbers (e.g., 5–10–15, 25–50–75).

Considering a broader range of sequences would also enable investigating whether the reverse distance effect operates differently across different sequence types. For instance, the reverse distance effect reportedly appears stronger in double-digit sequences than in single-digit sequences (e.g., Gattas et al., 2021; Lyons & Ansari, 2015). This difference is notable because it initially seems inconsistent with the view that the reverse distance effect results from consecutive sequences being facilitated due to being more familiar. This is because, from this perspective, the reverse distance effect should arguably be weaker rather than stronger in double-digit sequences, as double-digit consecutive sequences are presumably less familiar than single-digit consecutive sequences.

The present study offers a potential explanation for these differences, however, by emphasising that the reverse distance effect likely reflects the relative difference in familiarity between the consecutive and non-consecutive sequences, rather than the familiarity of the consecutive sequences alone. Consequently, the reverse distance effect may be strengthened by either increasing the familiarity of the consecutive sequences or by decreasing the familiarity of the non-consecutive sequences. Conversely, it may be weakened by decreasing the familiarity of the consecutive sequences or by increasing the familiarity of the non-consecutive sequences. Therefore, although the double-digit reverse distance effect would be weakened by not including familiar consecutive sequences such as 1–2–3 and 2–3–4, it would also be strengthened by not including familiar non-consecutive sequences such as 1–3–5 and 2–4–6. Accordingly, stronger reverse distance effects in double-digit sequences do not necessarily contradict the familiarity perspective, because the strength of the reverse distance effect is expected to depend on the familiarity of the included sequences. Future research may test this explanation experimentally by replicating the present design using double-digit sequences.

In conclusion, the present study suggests that the presence of the reverse distance effect likely depends on the familiarity of the sequences being processed. In particular, highly familiar sequences such as 1–2–3 appear to play a pivotal role in whether or not a reverse distance effect is produced. A key takeaway from this study, therefore, is that order verification researchers should carefully consider the familiarity of the sequences being processed, rather than assuming that sequence types (e.g., consecutive/non-consecutive) represent homogeneous categories. Nonetheless, more research is needed to differentiate the effects of familiarity from those of numerical size, as well as to determine whether the present findings generalise to double-digit sequences.

## Supplementary Information

Below is the link to the electronic supplementary material.

Supplementary file1 (DOCX 188 KB)

## Data Availability

Data, materials, and analyses scripts for this study are accessible via the Open Science Framework: osf.io/djsxf.

## References

[CR1] Attout, L., Noël, M.-P., & Majerus, S. (2014). The relationship between working memory for serial order and numerical development: A longitudinal study. *Developmental Psychology,**50*(6), 1667–1679. 10.1037/a0036496 24684717 10.1037/a0036496

[CR2] Banks, W. P. (1977). Encoding and processing of symbolic information in comparative judgments. In G. H. Bower (Ed.), *Psychology of learning and motivation* (Vol. 11, pp. 101–159). Academic Press. 10.1016/S0079-7421(08)60476-4

[CR3] Bourassa, A. (2014). *Numerical Sequence Recognition: Is Familiarity or Ordinality the Primary Factor in Performance?* [MA thesis, Carleton University]. https://curve.carleton.ca/282e5db5-4f36-4e5f-93fa-7a47bbaf5640

[CR4] Brunner, C., Schadenbauer, P., Schröder, N., Grabner, R. H., & Vogel, S. E. (2024). Electrophysiological correlates of symbolic numerical order processing. *PLoS ONE,**19*(3), e0301228. 10.1371/journal.pone.0301228 38512938 10.1371/journal.pone.0301228 PMC10956805

[CR5] Decarli, G., Sella, F., Lanfranchi, S., Gerotto, G., Gerola, S., Cossu, G., & Zorzi, M. (2023). Severe developmental dyscalculia is characterized by core deficits in both symbolic and nonsymbolic number sense. *Psychological Science,**34*(1), 8–21. 10.1177/09567976221097947 36282938 10.1177/09567976221097947

[CR6] Devlin, D., Moeller, K., Reynvoet, B., & Sella, F. (2022). A critical review of number order judgements and arithmetic: What do order verification tasks actually measure? *Cognitive Development,**64*, 101262. 10.1016/j.cogdev.2022.101262

[CR7] Devlin, D., Moeller, K., Xenidou-Dervou, I., Reynvoet, B., & Sella, F. (2023). Concepts of order: Why is ordinality processed slower and less accurately for non-consecutive sequences? *Quarterly Journal of Experimental Psychology*. 10.1177/17470218231220912 10.1177/17470218231220912 PMC11295408 38053316

[CR8] Devlin, D., Moeller, K., Xenidou-Dervou, I., Reynvoet, B., & Sella, F. (2024). Familiar sequences are processed faster than unfamiliar sequences, even when they do not match the count-list. *Cognitive Science,**48*(7), e13481. 10.1111/cogs.13481 38980993 10.1111/cogs.13481

[CR9] Dubinkina, N., Sella, F., & Reynvoet, B. (2021). Symbolic number ordering and its underlying strategies examined through self-reports. *Journal of Cognition,**4*(1), 25. 10.5334/joc.157 33954276 10.5334/joc.157 PMC8051157

[CR10] Dubinkina, N., Sella, F., Vanbecelaere, S., & Reynvoet, B. (2023). Symbolic number ordering strategies and math anxiety. *Cognition and Emotion*. 10.1080/02699931.2023.2175795 36757771 10.1080/02699931.2023.2175795

[CR11] Gattas, S. U., Bugden, S., & Lyons, I. M. (2021). Rules of order: Evidence for a novel influence on ordinal processing of numbers. *Journal of Experimental Psychology: General,**150*(10), 2100–2116. 10.1037/xge0001022 33818119 10.1037/xge0001022

[CR12] Gilmore, C., & Batchelor, S. (2021). Verbal count sequence knowledge underpins numeral order processing in children. *Acta Psychologica,**216*, 103294. 10.1016/j.actpsy.2021.103294 33838444 10.1016/j.actpsy.2021.103294

[CR13] Goffin, C., & Ansari, D. (2016). Beyond magnitude: Judging ordinality of symbolic number is unrelated to magnitude comparison and independently relates to individual differences in arithmetic. *Cognition,**150*, 68–76. 10.1016/j.cognition.2016.01.018 26851638 10.1016/j.cognition.2016.01.018

[CR14] Hutchison, J. E., Ansari, D., Zheng, S., De Jesus, S., & Lyons, I. M. (2022). Extending ideas of numerical order beyond the count-list from kindergarten to first grade. *Cognition,**223*, 105019. 10.1016/j.cognition.2022.105019 35121431 10.1016/j.cognition.2022.105019

[CR15] Jeffreys, H. (1961). *Theory of probability* (3rd ed.). Oxford University Press.

[CR16] Leth-Steensen, C., & Marley, A. A. J. (2000). A model of response time effects in symbolic comparison. *Psychological Review,**107*(1), 62–100. 10.1037/0033-295X.107.1.162 10687403

[CR17] Lyons, I. M., & Ansari, D. (2015). Numerical order processing in children: From reversing the distance-effect to predicting arithmetic. *Mind, Brain, and Education,**9*(4), 207–221. 10.1111/mbe.12094

[CR18] Lyons, I. M., & Beilock, S. L. (2011). Numerical ordering ability mediates the relation between number-sense and arithmetic competence. *Cognition,**121*(2), 256–261. 10.1016/j.cognition.2011.07.009 21855058 10.1016/j.cognition.2011.07.009

[CR19] Lyons, I. M., & Beilock, S. L. (2013). Ordinality and the nature of symbolic numbers. *Journal of Neuroscience,**33*(43), 17052–17061. 10.1523/JNEUROSCI.1775-13.2013 24155309 10.1523/JNEUROSCI.1775-13.2013 PMC6618433

[CR20] Lyons, I. M., Price, G. R., Vaessen, A., Blomert, L., & Ansari, D. (2014). Numerical predictors of arithmetic success in grades 1–6. *Developmental Science,**17*(5), 714–726. 10.1111/desc.12152 24581004 10.1111/desc.12152

[CR21] Morey, R. D., Rouder, J. N., Jamil, T., Urbanek, S., Forner, K., & Ly, A. (2022). *BayesFactor: Computation of Bayes Factors for Common Designs* (Version 0.9.12–4.4) [Computer software]. https://CRAN.R-project.org/package=BayesFactor

[CR22] Morsanyi, K., Peters, J., Battaglia, E., Sasanguie, D., & Reynvoet, B. (2024). The causal role of numerical and non-numerical order processing abilities in the early development of mathematics skills: Evidence from an intervention study. *Current Research in Behavioral Sciences,**6*, 100144. 10.1016/j.crbeha.2023.100144

[CR23] Morsanyi, K., van Bers, B. M. C. W., O’Connor, P. A., & McCormack, T. (2018). Developmental dyscalculia is characterized by order processing deficits: Evidence from numerical and non-numerical ordering tasks. *Developmental Neuropsychology,**43*(7), 595–621. 10.1080/87565641.2018.1502294 30058838 10.1080/87565641.2018.1502294

[CR24] Orrantia, J., Muñez, D., Matilla, L., Sanchez, R., San Romualdo, S., & Verschaffel, L. (2019). Disentangling the mechanisms of symbolic number processing in adults’ mathematics and arithmetic achievement. *Cognitive Science*. 10.1111/cogs.12711 30648799 10.1111/cogs.12711

[CR25] Peirce, J., Gray, J. R., Simpson, S., MacAskill, M., Höchenberger, R., Sogo, H., Kastman, E., & Lindeløv, J. K. (2019). PsychoPy2: Experiments in behavior made easy. *Behavior Research Methods,**51*(1), 195–203. 10.3758/s13428-018-01193-y 30734206 10.3758/s13428-018-01193-y PMC6420413

[CR26] Pinhas, M., Buchman, C., Lavro, D., Mesika, D., Tzelgov, J., & Berger, A. (2015). The neural signatures of processing semantic end values in automatic number comparisons. *Frontiers in Human Neuroscience*. 10.3389/fnhum.2015.00645 26640436 10.3389/fnhum.2015.00645 PMC4661242

[CR27] Pinhas, M., & Tzelgov, J. (2012). Expanding on the mental number line: Zero is perceived as the “smallest.” *Journal of Experimental Psychology: Learning, Memory, and Cognition,**38*(5), 1187–1205. 10.1037/a0027390 22369255 10.1037/a0027390

[CR28] R Core Team. (2020). *R: A language and environment for statistical computing. R Foundation for Statistical Computing, Vienna, Austria. URL *https://www.R-project.org/*.* [Computer software].

[CR29] RStudio Team. (2022). *RStudio: Integrated Development Environment for R. RStudio, PBC, Boston, MA URL *http://www.rstudio.com/*.* [Computer software].

[CR30] Sasanguie, D., & Vos, H. (2018). About why there is a shift from cardinal to ordinal processing in the association with arithmetic between first and second grade. *Developmental Science,**21*(5), e12653. 10.1111/desc.12653 29417697 10.1111/desc.12653

[CR31] Sella, F., Sasanguie, D., & Reynvoet, B. (2020). Judging the order of numbers relies on familiarity rather than activating the mental number line. *Acta Psychologica,**204*, 103014. 10.1016/j.actpsy.2020.103014 32004925 10.1016/j.actpsy.2020.103014

[CR32] Slipenkyj, M., Hutchison, J., Ansari, D., Lyons, I. M., & Bugden, S. (2024). Ordinal processing differences between children with persistent dyscalculia and typically performing children. *Canadian Journal of Experimental Psychology / Revue Canadienne De Psychologie Expérimentale*. 10.1037/cep0000343 39207377 10.1037/cep0000343

[CR33] Sommerauer, G., Graß, K.-H., Grabner, R. H., & Vogel, S. E. (2020). The semantic control network mediates the relationship between symbolic numerical order processing and arithmetic performance in children. *Neuropsychologia,**141*, 107405. 10.1016/j.neuropsychologia.2020.107405 32087204 10.1016/j.neuropsychologia.2020.107405

[CR34] Vogel, S. E., Faulkenberry, T. J., & Grabner, R. H. (2021). Quantitative and qualitative differences in the canonical and the reverse distance effect and their selective association with arithmetic and mathematical competencies. *Frontiers in Education*. 10.3389/feduc.2021.655747

[CR35] Vos, H., Gevers, W., Reynvoet, B., & Xenidou-Dervou, I. (2021). Ordinality: The importance of its trial list composition and examining its relation with adults’ arithmetic and mathematical reasoning. *Quarterly Journal of Experimental Psychology,**74*(11), 1935–1952. 10.1177/17470218211016794 10.1177/17470218211016794 PMC8450998 33899600

[CR36] Vos, H., Sasanguie, D., Gevers, W., & Reynvoet, B. (2017). The role of general and number-specific order processing in adults’ arithmetic performance. *Journal of Cognitive Psychology,**29*(4), 469–482. 10.1080/20445911.2017.1282490

[CR37] Wong, B., Bull, R., Ansari, D., Watson, D. M., & Liem, G. A. D. (2022). Order processing of number symbols is influenced by direction, but not format. *Quarterly Journal of Experimental Psychology,**75*(1), 98–117. 10.1177/17470218211026800 10.1177/17470218211026800 34092147

[CR38] Xu, C., LeFevre, J.-A., Burr, S. D. L., Maloney, E. A., Wylie, J., Simms, V., Skwarchuk, S.-L., & Osana, H. P. (2023). A direct comparison of two measures of ordinal knowledge among 8-year-olds. *Journal of Numerical Cognition,**9*(2), 253–267. 10.5964/jnc.10201

