# Supplementary material for: The presence of the reverse distance effect depends on the familiarity of the sequences being processed
Source: Psychol Res. 2025 Feb 28;89(2):58. doi: 10.1007/s00426-025-02090-8 (PMC11870951; doi:10.1007/s00426-025-02090-8)
Supplement: Supplementary file 1 — Supplementary file1 (DOCX 188 KB) [file 426_2025_2090_MOESM1_ESM.docx]

**Supplementary materials**

**Order verification task instructions**

In all three experiments, the order verification task began with the following instructions:

“On screen, you will be presented with some sequences. You are asked to indicate whether they are “in order” or not. Ordered sequences can be either consecutive (e.g.,. 1-2-3) or non-consecutive (e.g., 2-4-6). If a sequence is in order, press the “P” key. If a sequence is not in order, press the "Q” key.”

**Arithmetic verification task: method**

The arithmetic verification task followed the same general format as the order verification task but, instead of number sequences, arithmetic statements (e.g., 7 + 2 = 9) were presented in the centre of the screen. Participants were instructed to indicate whether or not these statements were correct using the “P” and “Q” keys on a QWERTY keyboard. Items remained on screen until a key press was registered. This was followed by a blank screen and then the next sequence (see Figure S1 for example and timings).

This task included 32 addition and 32 multiplication problems selected and adapted from an arithmetic task developed by the Educational Neuroscience Laboratory at the University of Graz (Schillinger et al., 2018; Vogel et al., 2017, 2019, 2021) and based on the French kit test (French et al., 1963). For each correct item (e.g., 2 × 6 = 12), we also included a corresponding incorrect item (e.g., 2 × 6 = 8). Half of these incorrect items were operator errors (e.g., 4 × 6 = 10) and half were operand errors (e.g., 8 × 5 = 45) (for full list of items, see Table S1). Accuracy and response times were recorded for each trial. Additionally, participants first completed ten randomly selected practice trials with on-screen feedback provided. No feedback was given during the critical trials. Overall, this task lasted approximately four minutes.


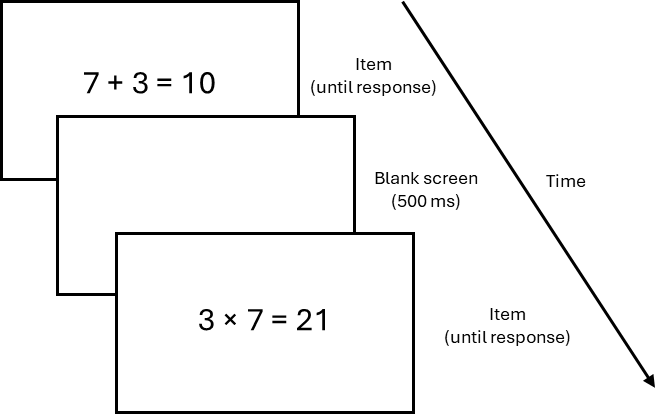


*Figure S1.* Example and timings of the order verification task

**Arithmetic verification task: results**

As an exploratory analysis, we also considered the association between response times on the order verification task and response times on the arithmetic verification task.

Each participant completed a total of 64 arithmetic verification trials, resulting in 5,885 responses recorded across 92 participants. Mean accuracy across all participants was 94.17%. Because we were only interested in correct responses to true arithmetic statements, we first removed all responses to false statements (n = 2,944; 50%) as well as all incorrect responses (n = 139; 4.72%). We then applied the same criteria for excluding extreme values as used for the order verification analysis. Accordingly, we removed responses that were longer than the mean plus three standard deviations (mean = 2,074 ms, *SD* = 2,498 ms) (n = 15; 0.53%). Again, there were no responses shorter than 200 ms. Following this trimming procedure, the distribution was still positively skewed (skewness = 2.35) and leptokurtic (kurtosis = 13.09). Therefore, to account for this, we considered only median response times for each participant in the subsequent analysis.

We then calculated correlations between response times on the order verification task and response times on the arithmetic verification task. This revealed a moderate positive correlation whereby faster responses on the order verification task were associated with faster responses on the arithmetic verification task, *r*(90) = .60, *p* < .001.


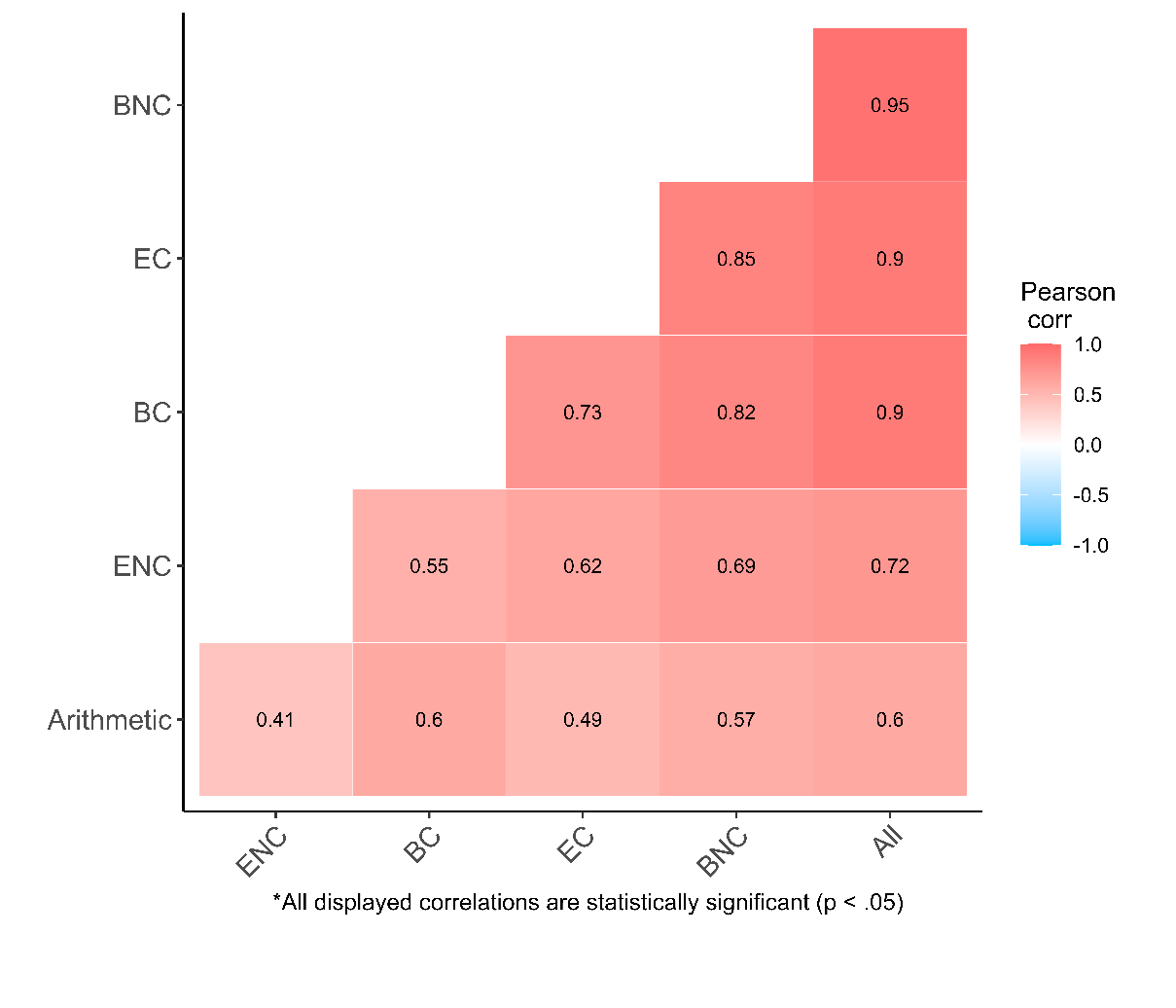


*Figure S2.* Correlations between arithmetic and order verification response times

After this, we considered the four sequence types individually: enhanced consecutive, enhanced non-consecutive, balanced consecutive, and balanced non-consecutive (all correlations are displayed in Figure S2). When considering only the enhanced familiarity condition, the association with arithmetic appeared marginally stronger for consecutive (.49) compared to non-consecutive sequences (.41); however, this difference was not statistically significant (Steiger’s *z = .94, p* = .345). When considering only the balanced familiarity condition, the correlation appeared comparable between consecutive (.60) and non-consecutive sequences (.57); this difference was also not statistically significant (Steiger’s *z = .61, p* = .540).

**Arithmetic verification task: discussion**

In experiment 1, we explored the association between order verification and arithmetic. Consistent with previous studies (e.g., Attout et al., 2014; Vos et al., 2017), we found that faster response times on the order verification task were associated with better performance on the arithmetic task. This association, however, did not differ between consecutive and non-consecutive sequences in either the enhanced or balanced familiarity conditions. This arguably conflicts with the familiarity perspective since one may have expected performance on the familiar sequences in each condition to show a stronger association with arithmetic than performance on the unfamiliar sequences. This is because, from the familiarity perspective, the association between order verification and arithmetic is thought to be mediated by the retrieval of familiar information from long-term memory (e.g., Devlin et al., 2022; Dubinkina et al., 2021). Therefore, more work is still needed to clarify the role of familiarity and memory retrieval in the association between order processing and arithmetic.

In this context, the present study was limited by only considering ascending and single-digit sequences as this resulted in relatively few highly familiar non-consecutive sequences being eligible for inclusion. Consequently, in the condition including the least familiar consecutive sequences and the most familiar non-consecutive sequences, the mean familiarity score was still slightly higher for consecutive compared to non-consecutive sequences (48.58 vs. 46.15). Accordingly, our approach to sequence selection prevented us from fully isolating the processing of highly familiar sequences from the processing of highly unfamiliar sequences. Therefore, to better investigate the role of familiarity in the association between order verification and arithmetic, future research may benefit from considering a broader range of sequences. For example, considering double-digit sequences may help identify more highly familiar non-consecutive sequences (e.g., 5-10-15, 25-50-75); this would thus potentially enable creating a condition in which the included non-consecutive sequences are considerably more familiar than the included consecutive sequences, which was not possible in the present design.

*Table S1*. All items included in the arithmetic verification task

| **problem** | **type** | **correct** | **error type** |
| --- | --- | --- | --- |
| 2 x 6 = 12 | multiplication | correct |  |
| 8 x 5 = 40 | multiplication | correct |  |
| 4 x 6 = 24 | multiplication | correct |  |
| 5 x 7 = 35 | multiplication | correct |  |
| 8 x 6 = 48 | multiplication | correct |  |
| 5 x 9 = 45 | multiplication | correct |  |
| 8 x 8 = 64 | multiplication | correct |  |
| 9 x 4 = 36 | multiplication | correct |  |
| 2 x 7 = 14 | multiplication | correct |  |
| 4 x 3 = 12 | multiplication | correct |  |
| 9 x 6 = 54 | multiplication | correct |  |
| 5 x 5 = 25 | multiplication | correct |  |
| 6 x 7 = 42 | multiplication | correct |  |
| 3 x 9 = 27 | multiplication | correct |  |
| 5 x 4 = 20 | multiplication | correct |  |
| 7 x 7 = 49 | multiplication | correct |  |
| 2 x 6 = 8 | multiplication | incorrect | operator |
| 8 x 5 = 45 | multiplication | incorrect | operand |
| 4 x 6 = 10 | multiplication | incorrect | operator |
| 5 x 7 = 40 | multiplication | incorrect | operand |
| 8 x 6 = 14 | multiplication | incorrect | operator |
| 5 x 9 = 54 | multiplication | incorrect | operand |
| 8 x 8 = 16 | multiplication | incorrect | operator |
| 9 x 4 = 32 | multiplication | incorrect | operand |
| 2 x 7 = 9 | multiplication | incorrect | operator |
| 4 x 3 = 16 | multiplication | incorrect | operand |
| 9 x 6 = 3 | multiplication | incorrect | operator |
| 5 x 5 = 30 | multiplication | incorrect | operand |
| 6 x 7 = 13 | multiplication | incorrect | operator |
| 3 x 9 = 36 | multiplication | incorrect | operand |
| 5 x 4 = 1 | multiplication | incorrect | operator |
| 7 x 7 = 42 | multiplication | incorrect | operand |
| 4 + 9 = 13 | addition | correct |  |
| 3 + 8 = 11 | addition | correct |  |
| 9 + 7 = 16 | addition | correct |  |
| 2 + 5 = 7 | addition | correct |  |
| 8 + 9 = 17 | addition | correct |  |
| 7 + 3 = 10 | addition | correct |  |
| 8 + 4 = 12 | addition | correct |  |
| 3 + 3 = 6 | addition | correct |  |
| 7 + 6 = 13 | addition | correct |  |
| 4 + 5 = 9 | addition | correct |  |
| 7 + 2 = 9 | addition | correct |  |
| 6 + 9 = 15 | addition | correct |  |
| 2 + 8 = 10 | addition | correct |  |
| 9 + 5 = 14 | addition | correct |  |
| 6 + 3 = 9 | addition | correct |  |
| 8 + 7 = 15 | addition | correct |  |
| 4 + 9 = 36 | addition | incorrect | operator |
| 3 + 8 = 12 | addition | incorrect | operand |
| 9 + 7 = 2 | addition | incorrect | operator |
| 2 + 5 = 6 | addition | incorrect | operand |
| 8 + 9 = 72 | addition | incorrect | operator |
| 7 + 3 = 9 | addition | incorrect | operand |
| 8 + 4 = 4 | addition | incorrect | operator |
| 3 + 3 = 7 | addition | incorrect | operand |
| 7 + 6 = 1 | addition | incorrect | operator |
| 4 + 5 = 8 | addition | incorrect | operand |
| 7 + 2 = 14 | addition | incorrect | operator |
| 6 + 9 = 16 | addition | incorrect | operand |
| 2 + 8 = 16 | addition | incorrect | operator |
| 9 + 5 = 13 | addition | incorrect | operand |
| 6 + 3 = 3 | addition | incorrect | operator |
| 8 + 7 = 16 | addition | incorrect | operand |

*Table S2*. Unique sequences in control conditions for experiments 2 and 3 (digits 1-9)

| Left | Centre | | Right | Type | Distance |
| --- | --- | --- | --- | --- | --- |
| 1 | 2 | 3 | | Ordered | 1 |
| 2 | 3 | 1 | | Non-ordered | 1 |
| 2 | 3 | 4 | | Ordered | 1 |
| 3 | 4 | 2 | | Non-ordered | 1 |
| 3 | 4 | 5 | | Ordered | 1 |
| 4 | 5 | 3 | | Non-ordered | 1 |
| 4 | 5 | 6 | | Ordered | 1 |
| 5 | 6 | 4 | | Non-ordered | 1 |
| 5 | 6 | 7 | | Ordered | 1 |
| 6 | 7 | 5 | | Non-ordered | 1 |
| 6 | 7 | 8 | | Ordered | 1 |
| 7 | 8 | 6 | | Non-ordered | 1 |
| 7 | 8 | 9 | | Ordered | 1 |
| 8 | 9 | 7 | | Non-ordered | 1 |

*Table S3*. Unique sequences in experiment 2 experimental condition (digits 0-8)

| Left | Centre | | Right | Type | Distance |
| --- | --- | --- | --- | --- | --- |
| 0 | 1 | 2 | | Ordered | 1 |
| 1 | 2 | 0 | | Non-ordered | 1 |
| 1 | 2 | 3 | | Ordered | 1 |
| 2 | 3 | 1 | | Non-ordered | 1 |
| 2 | 3 | 4 | | Ordered | 1 |
| 3 | 4 | 2 | | Non-ordered | 1 |
| 3 | 4 | 5 | | Ordered | 1 |
| 4 | 5 | 3 | | Non-ordered | 1 |
| 4 | 5 | 6 | | Ordered | 1 |
| 5 | 6 | 4 | | Non-ordered | 1 |
| 5 | 6 | 7 | | Ordered | 1 |
| 6 | 7 | 5 | | Non-ordered | 1 |
| 6 | 7 | 8 | | Ordered | 1 |
| 7 | 8 | 6 | | Non-ordered | 1 |

N.B. the experimental condition in experiment 2 contains almost the same sequences as the control condition, except 7-8-9 and 8-9-7 were replaced with 0-1-2 and 1-2-0.

*Table S4*. Unique sequences in repeated digits condition for experiment 3

| Left | Centre | | Right | Type | Distance |
| --- | --- | --- | --- | --- | --- |
| 1 | 2 | 3 | | Ordered | 1 |
| 2 | 3 | 1 | | Non-ordered | 1 |
| 1 | 2 | 1 | | Non-ordered | 1 |
| 2 | 3 | 4 | | Ordered | 1 |
| 3 | 4 | 2 | | Non-ordered | 1 |
| 2 | 3 | 2 | | Non-ordered | 1 |
| 3 | 4 | 5 | | Ordered | 1 |
| 4 | 5 | 3 | | Non-ordered | 1 |
| 3 | 4 | 3 | | Non-ordered | 1 |
| 4 | 5 | 6 | | Ordered | 1 |
| 5 | 6 | 4 | | Non-ordered | 1 |
| 4 | 5 | 4 | | Non-ordered | 1 |
| 5 | 6 | 7 | | Ordered | 1 |
| 6 | 7 | 5 | | Non-ordered | 1 |
| 5 | 6 | 5 | | Non-ordered | 1 |
| 6 | 7 | 8 | | Ordered | 1 |
| 7 | 8 | 6 | | Non-ordered | 1 |
| 6 | 7 | 6 | | Non-ordered | 1 |
| 7 | 8 | 9 | | Ordered | 1 |
| 8 | 9 | 7 | | Non-ordered | 1 |
| 7 | 8 | 7 | | Non-ordered | 1 |

N.B. the control condition in experiment 3 is the same as the control condition in experiment 2 (see Table S2). Furthermore, the repeated digits condition in experiment 3 includes twice as many unique non-ordered sequences as it does ordered sequences. To account for this, each non-ordered sequence was presented half the number of times as each ordered sequences. Therefore, participants were still presented with 50% ordered and 50% non-ordered sequences.

**References for supplementary materials**

Attout, L., Noël, M.-P., & Majerus, S. (2014). The relationship between working memory for serial order and numerical development: A longitudinal study. *Developmental Psychology*, *50*(6), 1667–1679. https://doi.org/10.1037/a0036496

Devlin, D., Moeller, K., Reynvoet, B., & Sella, F. (2022). A critical review of number order judgements and arithmetic: What do order verification tasks actually measure? *Cognitive Development*, *64*, 101262. https://doi.org/10.1016/j.cogdev.2022.101262

Dubinkina, N., Sella, F., & Reynvoet, B. (2021). Symbolic Number Ordering and its Underlying Strategies Examined Through Self-Reports. *Journal of Cognition*, *4*(1), 25. https://doi.org/10.5334/joc.157

French, J. W., Ekstrom, R. B., & Price, L. A. (1963). *Manual for kit of reference tests for cognitive factors*. Princeton, NJ: Educational Testing Service.

Schillinger, F. L., Vogel, S. E., Diedrich, J., & Grabner, R. H. (2018). Math anxiety, intelligence, and performance in mathematics: Insights from the German adaptation of the Abbreviated Math Anxiety Scale (AMAS-G). *Learning and Individual Differences*, *61*, 109–119. https://doi.org/10.1016/j.lindif.2017.11.014

Vogel, S. E., Faulkenberry, T. J., & Grabner, R. H. (2021). Quantitative and Qualitative Differences in the Canonical and the Reverse Distance Effect and Their Selective Association With Arithmetic and Mathematical Competencies. *Frontiers in Education*, *6*. https://www.frontiersin.org/article/10.3389/feduc.2021.655747

Vogel, S. E., Haigh, T., Sommerauer, G., Spindler, M., Brunner, C., Lyons, I. M., & Grabner, R. H. (2017). Processing the Order of Symbolic Numbers: A Reliable and Unique Predictor of Arithmetic Fluency. *Journal of Numerical Cognition*, *3*(2), 288–308. https://doi.org/10.5964/jnc.v3i2.55

Vogel, S. E., Koren, N., Falb, S., Haselwander, M., Spradley, A., Schadenbauer, P., Tanzmeister, S., & Grabner, R. H. (2019). Automatic and intentional processing of numerical order and its relationship to arithmetic performance. *Acta Psychologica*, *193*, 30–41. https://doi.org/10.1016/j.actpsy.2018.12.001

Vos, H., Sasanguie, D., Gevers, W., & Reynvoet, B. (2017). The role of general and number-specific order processing in adults’ arithmetic performance. *Journal of Cognitive Psychology*, *29*(4), 469–482. https://doi.org/10.1080/20445911.2017.1282490
